# Management of Pediatric Solid Organ Injuries

**DOI:** 10.3390/children11060667

**Published:** 2024-05-30

**Authors:** Bailey D. Lyttle, Regan F. Williams, Steven Stylianos

**Affiliations:** 1 Department of Surgery, University of Colorado School of Medicine and Children’s Hospital Colorado, 12631 East 17th Avenue, Room 6111, Aurora, CO 80045, USA; bailey.lyttle@cuanschutz.edu; 2 Department of Surgery, Le Bonheur Children’s Hospital, 49 North Dunlap Avenue, Second Floor, Memphis, TN 38105, USA; rfwillia@uthsc.edu; 3 Division of Pediatric Surgery, Columbia University Vagelos College of Physicians & Surgeons, Morgan Stanley Children’s Hospital, 3959 Broadway—Rm 204 N, New York, NY 10032, USA

**Keywords:** pediatric trauma, solid organ injury, spleen, liver, kidney, pancreas, surgery

## Abstract

Solid organ injury (SOI) is common in children who experience abdominal trauma, and the management of such injuries has evolved significantly over the past several decades. In 2000, the American Pediatric Surgical Association (APSA) published the first societal guidelines for the management of blunt spleen and/or liver injury (BLSI), advocating for optimized resource utilization while maintaining patient safety. Nonoperative management (NOM) has become the mainstay of treatment for SOI, and since the publication of the APSA guidelines, numerous groups have evaluated how invasive procedures, hospitalization, and activity restrictions may be safely minimized in children with SOI. Here, we review the current evidence-based management guidelines in place for the treatment of injuries to the spleen, liver, kidney, and pancreas in children, including initial evaluation, inpatient management, and long-term care, as well as gaps that exist in the current literature that may be targeted for further optimization of protocols for pediatric SOI.

## 1. Introduction

Trauma is the leading cause of death in children aged 1–14 years [1]. An estimated 10–15% of pediatric trauma patients experience abdominal injuries; the vast majority are due to blunt trauma, with the most common mechanisms including falls and motor vehicle accidents [2,3,4]. Children are at higher risk of intra-abdominal injuries due to their smaller size, less soft tissue to “pad” the transferred energy from a traumatic impact, and incompletely ossified bones leading to less protection of the underlying viscera [3]. Solid organ injury (SOI) refers to intra-abdominal organ injuries excluding hollow viscus organs and is estimated to occur in approximately 8800 children annually [5]. The most commonly injured solid organ in children is the liver, representing 44% of solid organ injuries, followed by the spleen (32%), kidney (18%), and pancreas (6%) [6]. While the mortality rate associated with isolated SOI is overall low, other factors, such as gastrointestinal injuries or the involvement of large blood vessels, can significantly increase the risk of death. Historically, SOI were diagnosed according to physical exam and clinical expertise, with exam findings that may indicate intra-abdominal injury (IAI) including abdominal tenderness, distension, guarding, abdominal wall ecchymosis or abrasions, tachycardia, and hypotension, though the sensitivity and specificity of physical exam findings for predicting IAI are overall poor [7,8]. 

**Figure 1:**
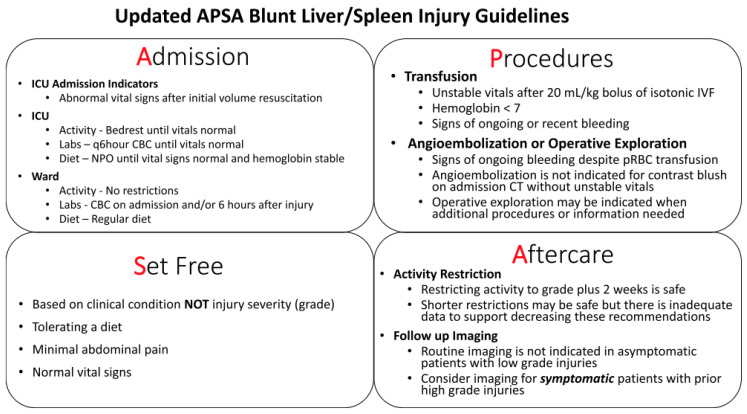
American Pediatric Surgical Association updated blunt liver/spleen guidelines. Updated American Pediatric Surgical Association (APSA) guidelines for the management of blunt liver and spleen injuries, designed as a “pocket card” with the easily recalled mnemonic “APSA” for Admission, Procedures, Set free, and Aftercare [13]. ICU = intensive care unit; q6hour = every six hours; CBC = complete blood count; NPO = nil per os; IVF = intravenous fluids; pRBC = packed red blood cells; CT = computed tomography.

Pediatric surgeons began considering nonoperative management (NOM) for select injuries as early as the 1960s [9,10,11]. Since then, particularly with the rise of computed tomography (CT) and the introduction of the American Association for the Surgery of Trauma (AAST) organ injury scale (Table 1), NOM has become the mainstay of treatment for hemodynamically stable pediatric patients with SOI [10]. In 2000, the American Pediatric Surgical Association (APSA) released guidelines for the management of blunt liver and/or spleen injuries (BLSIs), advocating for the minimization of intensive care unit (ICU) admissions and repeat imaging, decreased hospital length of stay (LOS), and reduced activity restrictions [12]. Since this initial release, APSA has re-evaluated and released updated guidelines for BLSI, overall following a similar pattern of recommending “less” in terms of in-hospital interventions and restrictions (Figure 1) [13,14]. Other organizations have released similar guidelines for SOI in an effort to create a more standardized approach to how these injuries are managed [15,16,17,18,19]. The purpose of this article is to review the current recommendations for the management of SOI in the pediatric population.

## 2. Initial Resuscitation

The initial assessment of pediatric trauma patients is protocolized by the American College of Surgeons (ACS) Advanced Trauma Life Support (ATLS) course material and follows the same principles as trauma management in adults, with the goal of the primary survey of the patient being the identification of hemodynamic instability or life-threatening conditions [20]. Consideration should be taken for the smaller body mass to body surface area ratio in children compared to adults, which makes children more susceptible to high-grade multisystem injuries and more highly sensitive to insensible fluid and heat loss [21]. Another important distinction in the pediatric patient is the ability to physiologically compensate following acute blood loss, with hypotension occurring in less than half of patients with BLSI who require early transfusion [22]. Numerous studies have sought to clearly define hemodynamic characteristics that may identify pediatric trauma patients in need of more aggressive intervention. Pediatric trauma patients who require early transfusion may be identified by an elevated pediatric age-adjusted shock index (SIPA) rather than hypotension alone, and SIPA has been further validated as a predictor of children with BLSI who will require transfusion, operative intervention, and ICU admission [23,24,25,26]. Additionally, recent evidence suggests that excessive crystalloid volume resuscitation is associated with poor outcomes, including prolonged ventilation and hospital LOS, in pediatric trauma patients, and over half of patients who receive multiple crystalloid boluses still ultimately require transfusion [27]. Crystalloid infusion should therefore be limited to a 20 mL/kg bolus, with persistent hemodynamic instability necessitating transfusion [5,13]. The transfusion of packed red blood cells is recommended for hemoglobin levels of less than 7.0 g/dL or for patients with signs of ongoing or recent bleeding (GRADE 1A recommendation, strong recommendation and high-quality evidence) [13,28]. Thromboelastography (TEG)-directed resuscitation allows providers to tailor blood product usage to specific deficiencies contributing to coagulopathy and has been associated with improved outcomes in children with blunt SOI, specifically when transfusing platelets and cryoprecipitate [29]. Early vasopressor use in SOI has been associated with an 11-fold increased risk of mortality and should be avoided, opting instead for adequate resuscitation and/or definitive management to address ongoing bleeding or shock [30].

## 3. Imaging

### 3.1. FAST and Contrast-Enhanced Ultrasound

While focused assessment with sonography for trauma (FAST) has known utility in the evaluation of adult patients with blunt abdominal trauma, its use in the evaluation of children has been less reliable due to low sensitivity and frequently missed injuries requiring intervention [15,31,32,33,34]. However, the Arizona-Texas-Oklahoma-Memphis-Arkansas Consortium (ATOMAC) demonstrated that a negative FAST is predictive of successful NOM for patients with BLSI [35]. Another recent study found that a positive FAST was an independent risk factor for IAI identified by CT, and Long et al. suggested that a positive FAST may aid in the prediction of hemodynamically unstable patients requiring early surgical intervention [34,36]. Contrast-enhanced ultrasound (CEUS) has been introduced as an imaging modality with more sensitivity than FAST alone and without the same radiation risks of CT, specifically with sensitivities and specificities of CEUS ranging from 85.7% to 100% and from 89 to 100%, respectively [37]. A meta-analysis of nine studies determined that CEUS had a sensitivity of 98.1% for the identification of parenchymal injuries in blunt abdominal trauma, though notably only three of the included studies included pediatric patients, and CEUS additionally failed to consistently identify injuries to smaller organs such as the adrenal gland that were easily identified on CT [38]. One small retrospective study including 59 patients found that CEUS completely agreed with CT in terms of identifying IAI [39]. Additional studies have similarly found that CEUS agrees with cross-sectional imaging and may offer some utility in the future as an alternative to CT to reduce oncologic risk in children, but additional studies are needed [40,41]. CT remains the imaging modality of choice for pediatric patients with blunt abdominal trauma and is recommended by radiological societies in both the United States and Europe [42,43].

### 3.2. Cross-Sectional Imaging

In general, hemodynamically unstable patients with abdominal trauma should forego initial cross-sectional imaging and be managed operatively to quickly identify and adequately control the source of instability. However, determining which hemodynamically stable pediatric trauma patients require CT requires careful consideration to avoid unnecessary radiation exposure, and numerous decision-making tools have been proposed over the years [44,45,46,47,48,49,50,51]. In a prospective study including 12,044 children from the Pediatric Emergency Care Applied Research Network (PECARN) dataset, a prediction rule was created to identify children with blunt torso trauma that are low risk for IAI based on the absence of signs and/or symptoms of thoracoabdominal trauma on physical exam and a Glascow Coma Scale (GCS) score greater than 13 [45]. Specific items included in the prediction rule were evidence of abdominal wall trauma or presence of a seatbelt sign, GCS, degree of abdominal tenderness, evidence of thoracic wall trauma, complaint of abdominal pain, absent or decreased breath sounds, and vomiting [45]. The prediction rule had a sensitivity of 92.5%, a specificity of 44.1%, and a negative predictive value (NPV) of 98.9% for any IAI and a sensitivity of 99%, a specificity of 56%, and an NPV of 99.9% for IAI requiring intervention (IAI-I), suggesting that those at very low risk for IAI based on physical exam alone could reasonably avoid CT [45]. In another prospective study including 2188 patients from 14 Level I pediatric trauma centers, Streck et al. created a five-item clinical prediction rule incorporating complaint of abdominal pain; abdominal wall trauma, distension, or tenderness; abnormal chest X-ray; AST > 200 U/L; and abnormal pancreatic enzymes, in which patients without any of the above were considered very low risk for IAI [46]. The same model was later validated in a PECARN study including 2435 patients, and both studies found the prediction rule to be highly sensitive for both IAI and IAI-I, with sensitivities of 98.4% and 100%, specificities of 38.1% and 34.7%, and NPVs of 99% and 100%, respectively (level 3–4 evidence) [46,47]. A single-institution prospective study performed in 2022 found that, after implementing a protocol based on the same five-item prediction rule criteria, there was a reduction in the number of CTs performed for abdominal trauma from 28% to 21% [49]. Similarly, Gaffley et al. evaluated the effects of implementing a guideline that also incorporated hemoglobin levels and hematuria and noted a significant reduction in CT usage, from 48.3% to 36.8%, with no missed injuries [48]. Hematuria is a common presenting symptom associated with renal injury and may indicate the need for additional imaging, though it may not necessarily predict the grade of renal injury [52]. According to the World Society of Emergency Surgery (WSES)–AAST guidelines for renal injury, hemodynamically stable pediatric patients with mild symptoms, no hematuria, and no other indications for CT imaging may undergo ultrasound (US) for initial imaging as an alternative to CT (GRADE 2A Recommendation, weak recommendation and high-quality evidence), but those with high-energy trauma and/or a drop in hematocrit with any degree of hematuria should undergo a contrast-enhanced CT including a delayed urographic phase [18]. Overall, while there is no overarching consensus on which specific laboratory values accurately predict which patients will have IAI, patients with a benign clinical exam who have normal laboratory values can safely forego abdominal imaging, while patients who present with clinical or laboratory abnormalities as described above will require further investigation.

Elevated levels of pancreatic enzymes, including amylase and lipase, may be used as an indication for abdominal imaging to evaluate for traumatic injury to the pancreas, as mentioned in the prospective study by Streck et al. [46]. However, it should be noted that neither amylase nor lipase levels may accurately predict the grade of pancreatic injury. A multicenter review of nine pediatric trauma centers found that while maximal amylase was predictive of pseudocyst formation, neither initial nor peak amylase/lipase were associated with grade of injury, indicating that repeated testing for these levels may not be beneficial in pediatric trauma patients [53]. For patients with suspected pancreatic parenchymal or ductal lesions, MRI is the preferred imaging [16]. Interestingly, a retrospective study comparing MRCP to CT in 21 children with blunt pancreatic injuries found that while MRCP outperformed CT in terms of duct visualization, it did not demonstrate a significant difference in terms of identifying duct disruption, which may instead require ERCP to properly visualize [54]. Overall, cross-sectional imaging guidelines vary between institutions depending on which variables are incorporated, and further standardization may be beneficial, particularly as guidance for adult surgeons who manage pediatric patients and tend to overuse CT [55].

## 4. Level of Care and Inpatient Management

### 4.1. ICU Admission

The original APSA recommendations suggested that hemodynamically stable patients with grades I–III BLSI were safe for floor admission given their lower rates of transfusions and operative interventions, whereas grade IV injuries necessitated ICU admission given their higher rates of transfusion and operative intervention [12]. Shortly thereafter, Mehall et al. published a prospective study evaluating a standardized algorithm for pediatric patients with BLSI in which patient care decisions were based on hemodynamic status rather than grade of injury, demonstrating that hemodynamically normal children could be safely managed without ICU-level monitoring [56]. Subsequent studies have demonstrated a significant reduction in hospital resource utilization when care decisions are based on hemodynamic status rather than injury grade, without subsequent adverse events [57,58]. In 2015, the ATOMAC guidelines provided a GRADE 1A recommendation (strong recommendation and high-quality evidence) for patient management based on hemodynamic status, and the APSA management guidelines have since been updated accordingly [13,14,28]. ICU admission is now recommended only for patients with persistently abnormal vital signs indicating hemodynamic instability despite adequate fluid resuscitation, regardless of grade [13]. While recent studies have shown that high-grade SOI remains an independent risk factor for ICU admission, many patients with high-grade SOI also demonstrate hemodynamic instability that would result in ICU admission regardless based on the updated guidelines [59,60]. 

The management of adolescent patients requires additional consideration given their physiologic similarity to adult patients. One study using the Trauma Quality Improvement Program’s (TQIP) annual datasets found that adolescents who are managed at an adult trauma center (ATC) are more likely to be admitted to the ICU and require blood transfusion, suggesting more aggressive management than those who presented to pediatric trauma centers (PTCs) [61]. Notably, despite the patients who were managed at ATCs having both higher injury severity scores (ISSs) and lower GCS, the odds of mortality were higher for adolescents managed at PTCs even when excluding patients with significant traumatic brain injuries (TBIs) [61]. While this finding requires further investigation, a less conservative approach, similar to the management of adult trauma patients, may be indicated in this specific patient population.

### 4.2. Phlebotomy

The frequency of phlebotomy was not addressed in the originally published APSA guidelines, though the updated APSA guidelines recommend a complete blood count (CBC) every six hours until vital signs have normalized [12,13]. Golden et al. proposed a pathway in which hemodynamically stable patients with BLSI would receive a single blood draw [62]. When 120 patients with BLSI were retrospectively analyzed to see how the proposed pathway would perform, they found that the new pathway would decrease blood draws by 70%, ICU admission by 65%, and hospital LOS by 37% [62]. A subsequent prospective study implementing a similar protocol found that most patients included were safely managed with a single blood draw, though more severely injured patients and those with active contrast extravasation on imaging were notably excluded from this analysis [63]. Patients in both studies who did require transfusion also demonstrated hemodynamic instability that would have otherwise removed them from the proposed pathway and therefore prevented a potential missed diagnosis that may be detected when following serial hemoglobin levels [62,63]. In a separate retrospective study, Acker et al. found that patients with BLSI who require surgical intervention or transfusion for ongoing bleeding do so within the first 24 h of presentation, suggesting that prolonged hemoglobin monitoring is likely to provide little clinical benefit [64]. Similarly, a prospective ATOMAC study demonstrated that while initial hemoglobin levels above 9.25 g/dL are associated with successful NOM, subsequent decreases in hemoglobin fail to predict which patients may require transfusion or surgical intervention [65]. Overall, hemoglobin levels correlate with hemodynamics in children with SOI, and it is safe to limit blood draws in children to situations in which evaluation is clinically indicated [66]. Noninvasive hemoglobin monitoring via continuous pulse CO-oximeter correlates closely with laboratory hemoglobin measurements and may offer another method for evaluating children with SOI at risk of delayed bleeding, though its utility may be limited for patients with shock, severe injuries, or darker skin [67,68].

## 5. Nonoperative and Operative Management

The management of SOI has evolved significantly over the last 50 years, and current recommendations are focused on NOM as the standard of care in hemodynamically stable children [11,15,18,69]. The APSA benchmark guidelines initially released in 2000 refer to the universal success amongst pediatric surgeons of NOM for splenic injury, which was subsequently extended to the management of other blunt SOI [12,70]. Currently, the vast majority of SOI are managed nonoperatively [71]. In a recent retrospective review of 1066 pediatric patients with abdominal trauma from both blunt and penetrating mechanisms, 96.5% were managed without surgical intervention [72]. The adoption of NOM initially failed to meet the expectations of published guidelines, particularly as the minority of children with traumatic injuries are treated at children’s hospitals [73]. Stylianos et al. released a retrospective review shortly after the publication of the original APSA guidelines comparing the care of children with splenic injuries at trauma and non-trauma centers and found that children were more likely to undergo operative intervention at non-trauma centers, with independent risk factors including higher grade splenic injuries, multiple injuries, or patients aged 15–19 years [73]. Previous studies have also shown that children and adolescents with splenic injuries who are admitted to ATCs are more likely to undergo surgical intervention than those admitted to PTCs, even when controlling for ISS and grade of injury, though the management of liver and kidney injuries appears to remain consistent between ATCs and PTCs [74,75,76,77].

Overall, the failure rate of NOM is low, and the majority of cases are due to reasons other than ongoing hemorrhage, such as injury to other organs [78]. For example, for splenic injuries specifically, the rate of delayed bleeding is estimated to be less than 0.2% [79]. Patients at risk of failing NOM include those with multiple injuries, higher ISS, and isolated pancreatic injuries, but patients who fail NOM usually do so within the first 24 h [80,81]. In fact, in a recent prospective study by Arbra et al., all interventions for SOI occurred within eight hours of presentation with a median time to intervention of two hours, and the rate of delayed bleeding specifically in splenic injuries is estimated to be less than 0.2% [82]. Even injuries secondary to penetrating trauma, which account for the minority of SOIs in children, can be managed nonoperatively in select cases, though high-grade injuries and those resulting from gunshot wounds are more likely to fail NOM [83]. When patients do undergo operative management, laparoscopy is only used in 7–16% of cases requiring surgical intervention, though this has increased over time, particularly due to progression at academic institutions [84,85]. Patients managed laparoscopically tend to be less injured and have a shorter hospital LOS but spend more time in the OR [85]. Additionally, conversion to laparotomy and overall mortality remain low for patients undergoing laparoscopy, at 18.6% and 0.4%, respectively [85].

### 5.1. Liver

Overall, pediatric liver injuries rarely require operative intervention, with around 3% of isolated liver injuries requiring laparotomy [80,86]. However, patients who exhibit hemodynamic instability and fail to respond to appropriate resuscitation will require operative intervention, according to a GRADE 2A recommendation from the WSES Guidelines [15]. The immediate goal of laparotomy in the setting of a liver injury is hemorrhage control, which, depending on the severity, can initially be accomplished via compression of the liver itself, compression of the aorta against the spine, or occlusion of the portal triad via the Pringle maneuver [87,88]. Parenchymal compression can be achieved with packing, with packs placed between the liver and diaphragm/abdominal wall as well as inferior to the liver [88]. In the setting of an unstable patient with concerns for hypothermia or metabolic derangements necessitating damage control surgery, the initial operation may conclude here, with the placement of a temporary abdominal closure and plans to return to the operating room within 24–72 h, once the patient has been adequately stabilized. If the patient has stabilized with packing, definitive management can be pursued. The posterior portion of the right lobe of the liver is the most common site of injury due to the presence of coronary ligaments that fix this portion of the liver in place, thereby exacerbating acceleration–deceleration injuries [89]. Proper hepatic artery injuries should be repaired, if possible, but selective ligation can be considered if repair is not feasible, with the understanding that ligation is associated with hepatic necrosis, abscess formation, and the development of biloma [15]. Portal vein injuries should be repaired primarily, and ligation should be avoided due to the risk of hepatic necrosis and bowel edema [15]. Devitalized hepatic tissue may be resected, though an anatomic hepatic resection is rarely indicated [15,87]. Extensive non-anatomic resections or anatomic resections can be more safely performed by experienced surgeons or those with hepatobiliary training [15,88]. Finally, intraoperative cholangiography may be employed to ensure that biliary drainage has not been compromised following any necessary suture ligation or resection [87].

### 5.2. Spleen

Operative management is also rarely necessary for splenic injuries in pediatric patients, with laparotomies occurring in around 4% of patients with isolated spleen injuries [80,86]. In fact, one retrospective study from 2016 estimated that the average pediatric surgeon would perform fewer than two splenectomies and fewer than one splenorrhaphy over a 30-year surgical career [90]. In accordance with the WSES guidelines for blunt splenic trauma, NOM is contraindicated for patients with other indications for a laparotomy and/or hemodynamic instability (GRADE 2A recommendation, weak recommendation and high-quality evidence) [17]. The presence of a concomitant traumatic brain injury has not been found to alter the outcomes of pediatric patients with splenic injuries and should not affect the decision to pursue NOM, which notably differs from the recommendations for adult patients [17,91]. Like liver trauma, operative splenic trauma is initially managed with hemorrhage control and packing of the abdomen. Packing may be left in place in order to pursue management with angiography, the use of which is further detailed below [5]. Otherwise, operative management options include splenectomy or splenorrhaphy. While not currently recommended in adults, partial splenectomy or splenorrhaphy are viable alternatives to splenectomy in the pediatric population and may offer preservation of some splenic function [17,92].

NOM is particularly favored for pediatric patients with splenic trauma due to the risk of overwhelming post-splenectomy infection (OPSI). While the rate of OPSI has declined over the years, the risk of mortality remains as high as 50% [11,93]. To prevent OPSI, post-splenectomy patients should undergo vaccination against encapsulated bacteria including *Streptococcus pneumoniae, Haemophilus influenzae* (type b), and *Neisseria meningitidis*, as well as annual vaccination against influenza [94]. Recommendations regarding the use of prophylactic antibiotics vary significantly, particularly regarding the duration of treatment. Prophylactic antibiotics are recommended in high-risk populations, including children less than five years of age and all patients for the first 1–2 years following splenectomy, across multiple guidelines [17,93,94]. Post-splenectomy patients may also be prescribed an emergency supply of antibiotics to be taken in the setting of unexplained fever or other constitutional symptoms concerning for systemic infection [17].

### 5.3. Kidney

Around 3% of isolated traumatic renal injuries in the pediatric population require laparotomy [80]. While indications for operative intervention differ across various guidelines, there is agreement throughout the literature that hemodynamic instability, despite appropriate resuscitation, is an absolute indication for intervention, particularly in the setting of penetrating trauma, though there is a role for angioembolization in select clinical scenarios [18,69,95]. Operative management is recommended in the setting of severe vascular injury, specifically avulsion of the renal pedicle without self-limiting bleeding, according to the WSES–AAST Guidelines (GRADE 1C recommendation, strong recommendation and low-quality evidence) [18]. While older studies have used the presence of devitalized renal tissue as a relative indication for surgical intervention, current WSES–AAST recommendations state that non-viable renal tissue is not an indication for operative intervention unless other indications for laparotomy are present [18,69]. Ureteropelvic disruption is more common in the pediatric population due to the presence of congenital anomalies and also requires operative intervention [95]. Intra-operatively, the goal is once again hemorrhage control, as well as renal salvage. If a retroperitoneal hematoma is identified intra-operatively in the setting of penetrating trauma, or if the hematoma is pulsatile or expanding in the setting of blunt trauma, exploration is indicated [96]. Vascular control is obtained by dissecting out and controlling the renal hilum prior to opening the Gerota’s fascia, after which renorrhaphy can be performed if the tissue appears viable [95,96]. In the setting of devitalized tissue, partial or total nephrectomy is indicated depending on the extent of the damage, ensuring a watertight closure of the collecting system via an omental pedicle flap or renal fat bolster if a partial nephrectomy is performed [96]. Urine leaks secondary to renal injury are overall uncommon [97]; only half require some sort of intervention, which is most commonly stent placement, but the presence of a urine leak is a risk factor for requiring surgical intervention and is associated with increased morbidity [97,98]. Renal artery occlusion is another rare complication of renal trauma, and while data are overall limited, there have been reports of successful management with endovascular intervention [99,100].

### 5.4. Pancreas

Pancreatic injuries are uncommon in pediatric abdominal trauma [6]. Given both the rarity and complexity of pancreatic injuries, the WSES–AAST recommend that these patients should be managed exclusively at trauma centers (GRADE 1C recommendation, strong recommendation and low-quality evidence) [16,101]. Endoscopic retrograde cholangiopancreatography (ERCP) can be both diagnostic and therapeutic, even in the early period following pancreatic trauma, but, overall, its utility in children remains controversial [16]. One retrospective review of 22 trauma centers (with ERCP available at 14/22 centers) found that while ERCP was reported to alter management or subjectively improve outcomes in half the patients, there were no significant differences in outcomes or time to recovery when comparing patients managed nonoperatively who underwent ERCP versus those who were just observed [102]. In a similar single-institution review of 31 patients, ERCP was considered to be helpful in the direction of clinical decision-making in 60% of patients who underwent the procedure, but, again, there was little difference when these patients were compared to those who did not undergo ERCP [103]. Endoscopic treatment with stenting across the disrupted pancreatic duct may be a feasible option in select patients with high-grade injuries, but many patients ultimately still require surgical intervention for persistent ductal leak or peripancreatic fluid collection [104,105]. Overall, these results suggest that while ERCP may provide some therapeutic benefit in select patients, it functions more aptly as a tool for directing the next steps in care.

In general, decision making regarding operative management for pancreatic trauma is guided by the presence, location, and degree of duct involvement [6]. However, NOM of pancreatic injuries is both more common and more successful in pediatric patients compared to adults, even in higher grade (III-V) injuries, with reported success rates as high as 89% [16]. One study of 424 patients from the National Trauma Data Bank (NTDB) suggested that pancreatic injuries managed nonoperatively had equivalent or improved outcomes compared to those requiring upfront or delayed surgical intervention, though the NTDB notably does not distinguish grade to identify patients with or without ductal injury [106]. This study may also be identifying patients who failed NOM as delayed operations, as the NTDB does not provide any distinction explaining surgical timing, so these results should be interpreted cautiously [107]. Patients who undergo NOM are more likely to form peripancreatic fluid collections compared to patients managed operatively [106,108,109]. One multi-institutional review also found that patients undergoing NOM experienced overall longer hospitalizations (17.5 vs. 12.6 days), time to goal oral feeds (15.1 vs. 7.8 days), and time to complete resolution (38.6 vs. 22.6 days) compared to those who underwent surgical intervention [108]. A separate institution found that only one of seven patients with high-grade pancreatic injuries could be successfully managed nonoperatively, though this institution notably does not offer ERCP as a diagnostic tool or adjunct to NOM [110]. Endocrine and exocrine function may be impaired following the NOM of traumatic pancreatic injury, but there is currently limited literature exploring this, and further studies are required [111]. When operative intervention is indicated, laparoscopic surgery is a feasible option in centers with an experienced team and may prevent a patient from requiring an open operation [112].

Peripancreatic fluid collections are common following pancreatic injuries and can be subdivided based on when they occur in relation to injury, with acute pancreatic fluid collections (APFCs) forming within four weeks and pseudocysts forming thereafter [113]. Overall, they are a relatively benign complication that often resolve spontaneously [109]. Rosenfeld et al. performed a multi-institution retrospective review of 100 pancreatic injuries in which 42% developed organized fluid collections and compared patients who were observed to those who underwent a drainage procedure upon identification of the collection [114]. Ultimately, there were no differences between the groups in terms of TPN use, hospital LOS, time to a regular diet, or the need for definitive drainage, and 70% of patients who were initially observed experienced spontaneous resolution, suggesting that this is a safe and effective strategy for pseudocyst management [114]. In a subset analysis of patients with larger collections measuring over seven centimeters in diameter, while these patients did have longer LOS and time to a regular diet than those with smaller collections, there were neither differences regarding the need for definitive procedures nor a difference in outcomes between patients with large collections who underwent observation versus drainage [114]. In all, APFCs and pseudocysts are likely safe to observe unless a patient is symptomatic, though larger collections will likely take longer to resolve and may benefit from drainage to prevent progression to a symptomatic collection requiring surgery.

## 6. Role of Angiography/Angioembolization

While the initial APSA benchmark guidelines did not provide any specific recommendations regarding the role of arterial embolization in BLSI, a systematic review performed by the APSA Outcomes and Evidence Based Practice Committee in 2019 recommended the selective use of angioembolization (AE) in the setting of ongoing bleeding with hemodynamic compromise as an adjunct to NOM for spleen, liver, and kidney injuries based on Level 3–4 evidence with an overall Grade D recommendation [14]. The committee also specified that prophylactic use of AE in the setting of a contrast blush is not indicated in the pediatric population, and the APSA guidelines have since been updated accordingly [12,13,14].

The current management of SOI regarding the use of angiography (AG) varies between adult and pediatric populations depending on the involved organ. For splenic injuries, specifically, the management of adult patients includes AE for hemodynamically stable patients with grade III lesions and/or the presence of contrast blush [17]. Similarly, in hemodynamically stable patients with liver injuries, AG/AE is considered the first-line intervention for adults presenting with a contrast blush, while children with similar injuries may be monitored [15]. However, multiple studies have suggested that children with similar injuries may be safely observed. In a retrospective study by Ingram et al., the few pediatric patients with BLSI that presented with contrast blush were more likely to require a higher level of care, transfusion, and surgical interventions, but these patients were overall more severely injured, and 80% of the patients with a blush present were still able to be managed nonoperatively [115]. Bansal et al. found no differences between pediatric patients that presented with or without a contrast blush, and, interestingly, contrast blush was present in only one of the six patients to undergo a splenectomy in their patient cohort [116]. An ATOMAC study from 2017 found that 37% of patients with BLSI that undergo AE still require surgical intervention [78]. The management of pediatric kidney injuries aligns more closely with adult management, with AE being used more selectively in both populations [18,19]. In all three organs, however, AE should be considered in pediatric patients with ongoing or delayed bleeding and no other indication for surgery as an adjunct to NOM rather than operative intervention [15,17,18,19,117].

Despite the current pediatric guideline recommendations, children managed by surgeons more familiar with adult trauma protocols may be at risk of additional unnecessary procedures. One study found that the odds of undergoing AE for pediatric patients are significantly higher when managed at an ATC rather than a PTC [118]. Isolated splenic injuries, specifically, incur a 9-fold higher risk of undergoing AE when managed at an ATC, without any improvement in splenic salvage rates [118]. More recent studies have found that the difference in management between ATCs and PTCs disappears with multivariate analysis, while other factors such, as age and ISS, more consistently predict the likelihood of a pediatric patient undergoing AE at an ATC, which may suggest a shift in how adult surgeons are now approaching pediatric patients [119,120]. One institution described their experience of managing blunt splenic trauma after extending the adult AE protocol to adolescent patients aged 15–17 years, in which AE was mandated for all patients with injuries grade III or higher, active contrast extravasation, or pseudoaneurysm formation [121]. Splenic preservation was achieved in 90% of cases and NOM was successful in 98% [121]. The WSES guidelines have subsequently recommended that adolescents over the age of 15 with splenic injuries should be managed in accordance with current adult protocols (Grade 1C recommendation, strong recommendation and low-quality evidence), though this has not been carried forward by other organizations [17].

## 7. Activity Restrictions and Hospital Discharge

### 7.1. Bedrest

While specific recommendations related to bedrest were not included in the original APSA guidelines, a “grade plus one day” recommendation was provided regarding hospital LOS, which some have previously also used as the expectation for bedrest [12]. In 2011, St. Peter et al. performed a prospective study including 110 patients with BLSI with a mean injury grade of 2.6 demonstrating the safety of an abbreviated bedrest protocol (ABRP), in which grades I and II injuries were managed with one night of bedrest and grades III and higher with two nights of bedrest [122]. These results were later validated in a study from the same group, including 199 patients managed with the same ABRP, that demonstrated similar results [123]. In a retrospective review of over 22,000 patients from the Kids’ Inpatient Database, Dodgion et al. found that the same protocol would result in a LOS reduction of 1.7 hospital days per patient if utilized [2]. In all, an ABRP appears to be safe in children with BLSI and results in decreased hospital resource utilization. The ATOMAC guidelines published in 2015 recommend an abbreviated period of bedrest of one day or less (GRADE 1A recommendation, strong recommendation and high-quality evidence), and the current APSA guidelines recommend bedrest until vital signs have normalized [28].

Recommendations specific to renal injuries are not included in the ATOMAC or APSA guidelines. A prospective study by Graziano et al. found that the mean time to ambulation for patients with renal injuries was 1.5 days overall and 0.8 days when excluding patients with multiple injuries that otherwise limited ambulation [124]. No patients developed delayed bleeding requiring transfusion or intervention, suggesting that a limited bedrest approach is likely also safe in renal injury. Additionally, the majority of patients with renal trauma had persistent hematuria at follow-up appointments, suggesting that this finding is of little clinical utility when determining if a patient is safe for activity [124].

### 7.2. Activity Restrictions

The originally published APSA guidelines recommended a “grade plus two weeks” approach to activity restrictions in BLSI, determined based on what was safely done in at least 25% of the included patients [12]. A recent study that evaluated activity restriction adherence and 60-day outcomes in BLSI found that of the 366 patients who completed follow-up, 49 (13.6%) reported non-adherence [125]. There were no differences in terms of presentation to the ED or hospital readmission between adherent and non-adherent patients, even when performing a sub-analysis of high-grade injuries [125]. Accordingly, the updated APSA guidelines are in agreement with the originally published guidelines for activity restrictions, but note that lighter restrictions may be safe [13]. In fact, the most recent WSES follow-up guidelines for splenic trauma suggest that restrictions of four weeks are safe in pediatric patients regardless of injury grade, but further studies are still needed to optimize activity restrictions [126].

### 7.3. Hospital Discharge

As mentioned above, the 2000 APSA guidelines recommended a “grade plus one day” approach to hospital LOS for children with BLSI [12]. Over the years, it has become evident that this approach likely overutilizes hospital resources, and a 2014 study demonstrated that pediatric surgeons were already shortening LOS for BLSI despite the recommendations [2]. Since shifting to management based on hemodynamic status rather than injury grade, there have been significant reductions in LOS without increases in adverse events [28,59,127]. One study evaluating patients with BLSI managed at both ATCs and PTCs found that while hospital LOS has decreased at each of these centers over time, LOS remains higher than what would be expected based on current APSA guidelines and further reinforcement of guidelines within the national trauma network, and particularly at ATCs, may be necessary [77].

Recent studies have also suggested that there may be patients with low-grade SOI who could be safely observed and discharged from the emergency department [128,129,130]. In a recent prospective observational study, Plumblee et al. found that among the 148 patients with grade I or II SOI and no other major injuries, no patients required acute interventions, including transfusion, angioembolization, or operative intervention [129]. Another retrospective study of 1019 children from the ACS TQIP registry with low grade SOI and no other major injuries found that only 1.7% of patients required an intervention, and the most common intervention was notably angiography which, upon closer analysis, was unlikely to be indicated in these low-grade injuries [128]. The current APSA guidelines recommend hospital discharge based on clinical status rather than grade, and a patient is considered appropriate for discharge once vital signs are normalized and they are tolerating a regular diet with minimal abdominal pain [13].

## 8. Post-Discharge Care

### 8.1. Reimaging

Reimaging has not been routinely recommended for patients with SOI since the original APSA guidelines were published in 2000 [12,13]. In a secondary analysis following a multi-site prospective study of BLSI patients, Notrica et al. found that 6% underwent any sort of reimaging, and of these patients only 11% resulted in hospitalization or intervention, suggesting that a selective approach to reimaging SOI is likely safe [131]. Pseudoaneurysms (PSAs) following splenic injury are a concern that may prompt reimaging, particularly as reimaging and embolization of PSAs are common practice in adults [17]. One single-institution study reported a rate of PSA in children with splenic injuries to be as high as 17% and therefore recommended CEUS as follow-up imaging, but, as prior studies have reported much lower rates of PSA, these results may simply be due to the natural evolution of the injury rather than identification of a lesion requiring intervention [132,133,134,135]. In a systematic review, Martin et al. identified 45 cases of post-traumatic splenic PSA, and while the vast majority of these patients were clinically stable, most still underwent splenectomy, splenorrhaphy, or embolization due to the fear of delayed complications [134]. However, of the nine patients that were observed, there was only one instance of splenic rupture and no deaths [134]. Similarly, PSA appears to be equally uncommon following liver and kidney injuries and unlikely to require intervention [135]. Bile leak, or biloma, can rarely occur secondary to liver injuries grade III or higher and present with right upper quadrant pain, fever, and/or jaundice [88]. Appropriate imaging would include a right upper quadrant US and hepatobiliary iminodiacetic acid (HIDA) scan [88]. Overall, the most recent APSA guidelines recommend against routine imaging for low-grade BLSI injuries, with consideration for imaging in high-grade injuries only when patients are symptomatic [13]. For renal injuries, complications after discharge are overall rare, and follow-up imaging should be limited to those with moderate to severe (grades III–V) injuries [18]. For those that require follow-up imaging for both renal and pancreatic injuries, US or CEUS is the imaging modality of choice, with MRI being an option if cross-sectional imaging is indicated [16,18].

### 8.2. Additional Follow-Up

There are currently limited specific recommendations regarding when children with SOI require specific follow-up, with most providers currently making these decisions based on a constellation of injuries and institutional protocols. Pediatric trauma patients in general are at risk of emotional and functional limitations following injury and hospitalization, and behavioral health screening while in hospital for additional post-discharge support services may be beneficial [136]. Current WSES guidelines specific to splenic injuries suggest that the follow-up should be focused on the patient’s psychological response to their injuries, with consideration for reimaging only when clinically indicated [126]. 

For patients with renal injuries, ischemia from the injury itself or secondary vascular lesions can result in renin-mediated post-traumatic hypertension in approximately 4.2% of pediatric patients [19]. While the risk is low overall, current guidelines recommend routine blood pressure checks following renal trauma as a low cost and noninvasive intervention to prevent long-term adverse effects secondary to hypertension [19]. 

## 9. Discussion

The management of pediatric SOI is a topic of great interest, with numerous groups dedicating time and resources to the creation of reliable management guidelines. From the first introduction of NOM for splenic injuries to the most recently published APSA management guidelines, strategies for approaching SOI in children have tended towards minimizing unnecessary and invasive procedures. While the current literature discussing the management of pediatric SOI is overall relatively comprehensive, there do remain some gaps towards which future research may be directed. A recent systematic review of various clinical practice guidelines related to pediatric SOI found a paucity of recommendations specifically related to thromboprophylaxis, pain management, and nutritional support [137]. Recommendations related to thromboprophylaxis are especially needed, as SOI is a known risk factor for venous thromboembolism, yet thromboprophylaxis may be avoided due to concerns for delayed bleeding or differences in management compared to adult patients [138]. Furthermore, areas that have been more extensively studied, such as the utility of hemoglobin monitoring in pediatric patients with SOI, will still benefit from further research as technology evolves. For example, hemoglobin monitoring via continuous pulse CO-oximeter has been proposed as a noninvasive method of observing SOI and correlates with both isolated hemoglobin measurements and trends [67,68]. This method may offer an option for earlier discharge, with an at-home continuous hemoglobin monitor that sends reports directly to the provider or that can be remotely accessed, and is an area for future studies as the care of SOI continues to evolve. 

One additional area for potential improvement noted throughout this review is the difference in management and outcomes of children treated at ATCs compared to PTCs, including the use of imaging, ICU admissions, and interventions. Interestingly, one center previously accredited as an ATC that treated children described their experience following official PTC accreditation and noted a significant decrease in rates of surgical intervention for splenic injuries [139]. While the rates of operation for SOI in pediatric patients have decreased over the last decade generally, the rate at which surgical intervention decreased at this single institution exceeded expectations compared to national data [2,139]. This center’s experience may therefore be at least partially attributed to a transition to more pediatric-oriented protocols, and future studies could further elucidate this relationship if other centers undergo similar transitions from ATC to PTC accreditation. 

The dissemination of pediatric guidelines to participating trauma centers also remains essential to ensuring the appropriate care of children with SOI by providers who infrequently treat children. Further research is specifically needed regarding the management of adolescent patients, as numerous studies demonstrate differences in the care of this population [61,119,121]. While NOM has become more common in adults, discrepancies in management still exist, particularly regarding ICU admissions and the use of AE, with a potential mortality benefit for adolescents who are managed more aggressively [61]. Determining where adolescent patients fall in the physiologic spectrum is key to creating more tailored guidelines regarding if adolescents with SOI should be approached from a pediatric or adult perspective.

Finally, inconsistency in the management of pediatric trauma patients across institutions still exists despite the numerous guidelines currently available. A study by Vogel et al. found great variability in the initial management of blunt abdominal trauma between 14 pediatric trauma centers, specifically regarding the utilization of laboratory tests and imaging [140]. These findings unveil a potential target for quality improvement in the treatment of children with abdominal trauma who may benefit from more specific protocolization. As technology continues to advance and artificial intelligence plays a larger role in how research questions are approached, this may offer new ways to identify critically ill children. For example, Shahi et al. utilized deep learning–based models to predict the need for massive transfusion, failure of NOM, and mortality in children with blunt SOI using data available within the first four hours of admission with high sensitivity and accuracy [141]. Furthermore, the review by Yanchar et al. found that only one-third of current recommendations were based on at least moderate-quality evidence [137]. This lack of high-quality evidence poses significant challenges to creating and disseminating definitive guidelines related to the management of pediatric trauma patients. High-quality data, particularly from prospective studies and randomized controlled trials, when applicable, are still needed to optimize the care of children with SOI.

## 10. Conclusions

In conclusion, the management of solid organ injury in pediatric patients has evolved significantly over the years. While there are numerous facets of patient management that still lack a consensus, the overarching recommendations for pediatric patients with solid organ injuries focus on minimizing interventions and hospital stays in hemodynamically stable children. 

## Figures and Tables

**Table 1:** Injury scoring scales for solid organs.

Grade	Description of Injury
Spleen	
I	Capsular tear, subcapsular hematoma <10% surface area, laceration < 1 cm
II	Subcapsular hematoma 10–50% surface area or intraparenchymal < 5 cm, laceration 1–3 cm
III	Subcapsular hematoma >50% surface area or ruptured, intraparenchymal hematoma ≥ 5 cm, parenchymal laceration > 3 cm
IV	Vascular injury or active bleeding confined to capsule, parenchymal laceration involving segmental or hilar vessels with >25% devascularization
V	Vascular injury with active bleeding into peritoneum, hilar vascular injury with complete devascularization, shattered spleen
Liver	
I	Subcapsular hematoma <10% surface area, parenchymal laceration < 1 cm, capsular tear
II	Subcapsular hematoma 10–50% surface area, intraparenchymal hematoma <10 cm diameter, laceration 1–3 cm depth and ≤10 cm length
III	Subcapsular hematoma >50% surface area or expanding, ruptured hematoma, intraparenchymal laceration or hematoma > 10 cm, laceration >3 cm depth, vascular injury, contained active parenchymal bleeding
IV	Parenchymal disruption involving 25–75% of hepatic lobe, active bleeding into peritoneum
V	Parenchymal disruption involving >75% of hepatic lobe, juxtahepatic venous injury
Kidney	
I	Subcapsular hematoma, parenchymal contusion
II	Perirenal hematoma confined to Gerota fascia, parenchymal laceration ≤1 cm depth
III	Renal parenchymal laceration >1 cm depth without collecting system involvement, vascular injury or active bleeding contained within Gerota fascia
IV	Parenchymal laceration extending into urinary collection system with extravasation, renal pelvis lacearation, complete ureteropelvic disruption, segmental renal vascular injury, active bleeding into retroperitoneum or peritoneum, infarction due to vessel thrombosis
V	Main renal vascular laceartion or avulsion of hilum, devascularized kidney with active bleeding, shattered kidney
Pancreas	
I	Minor contusion or superficial laceration without duct injury
II	Major contusion or laceration without duct injury or tissue loss
III	Distal transection or parenchymal injury with duct injury
IV	Proximal transection or parenchymal injury involving ampulla
V	Massive disruption of pancreatic head

## References

[1] Centers for Disease Control and Prevention, National Center for Health Statistics National Vital Statistics System, Mortality 2018–2021 on CDC WONDER Online Database, Released in 2021. http://wonder.cdc.gov/ucd-icd10-expanded.html.

[2] Dodgion C.M., Gosain A., Rogers A., St Peter S.D., Nichol P.F., Ostlie D.J. (2014). National trends in pediatric blunt spleen and liver injury management and potential benefits of an abbreviated bed rest protocol. J. Pediatr. Surg..

[3] Gaines B.A. (2009). Intra-abdominal solid organ injury in children: Diagnosis and treatment. J. Trauma Acute Care Surg..

[4] Cooper A. (2020). Early Assessment and Management of Trauma. Holcomb and Ashcraft’s Pediatric Surgery.

[5] Notrica D.M. (2020). Abdominal and Renal Trauma. Holcomb and Ashcraft’s Pediatric Surgery.

[6] Notrica D. (2022). Evidence-based management of pediatric solid organ injury. Semin. Pediatr. Surg..

[7] Lynch T., Kilgar J., Al Shibli A. (2018). Pediatric Abdominal Trauma. Curr. Pediatr. Rev..

[8] Wegner S., Colletti J.E., Van Wie D. (2006). Pediatric blunt abdominal trauma. Pediatr. Clin. N. Am..

[9] Aronson D.Z., Scherz A.W., Einhorn A.H., Becker J.M., Schneider K.M. (1977). Nonoperative management of splenic trauma in children: A report of six consecutive cases. Pediatrics.

[10] Notrica D.M., Linnaus M.E. (2017). Nonoperative Management of Blunt Solid Organ Injury in Pediatric Surgery. Surg. Clin. N. Am..

[11] Stylianos S. (2019). To save a child’s spleen: 50 years from Toronto to ATOMAC. J. Pediatr. Surg..

[12] Stylianos S. (2000). Evidence-based guidelines for resource utilization in children with isolated spleen or liver injury. The APSA Trauma Committee. J. Pediatr. Surg..

[13] Williams R.F., Grewal H., Jamshidi R., Naik-Mathuria B., Price M., Russell R.T., Vogel A., Notrica D.M., Stylianos S., Petty J. (2023). Updated APSA Guidelines for the Management of Blunt Liver and Spleen Injuries. J. Pediatr. Surg..

[14] Gates R.L., Price M., Cameron D.B., Somme S., Ricca R., Oyetunji T.A., Guner Y.S., Gosain A., Baird R., Lal D.R. (2019). Non-operative management of solid organ injuries in children: An American Pediatric Surgical Association Outcomes and Evidence Based Practice Committee systematic review. J. Pediatr. Surg..

[15] Coccolini F., Coimbra R., Ordonez C., Kluger Y., Vega F., Moore E.E., Biffl W., Peitzman A., Horer T., Abu-Zidan F.M. (2020). Liver trauma: WSES 2020 guidelines. World J. Emerg. Surg..

[16] Coccolini F., Kobayashi L., Kluger Y., Moore E.E., Ansaloni L., Biffl W., Leppaniemi A., Augustin G., Reva V., Wani I. (2019). Duodeno-pancreatic and extrahepatic biliary tree trauma: WSES-AAST guidelines. World J. Emerg. Surg..

[17] Coccolini F., Montori G., Catena F., Kluger Y., Biffl W., Moore E.E., Reva V., Bing C., Bala M., Fugazzola P. (2017). Splenic trauma: WSES classification and guidelines for adult and pediatric patients. World J. Emerg. Surg..

[18] Coccolini F., Moore E.E., Kluger Y., Biffl W., Leppaniemi A., Matsumura Y., Kim F., Peitzman A.B., Fraga G.P., Sartelli M. (2019). Kidney and uro-trauma: WSES-AAST guidelines. World J. Emerg. Surg..

[19] Hagedorn J.C., Fox N., Ellison J.S., Russell R., Witt C.E., Zeller K., Ferrada P., Draus J.M. (2019). Pediatric blunt renal trauma practice management guidelines: Collaboration between the Eastern Association for the Surgery of Trauma and the Pediatric Trauma Society. J. Trauma Acute Care Surg..

[20] McFadyen J.G., Ramaiah R., Bhananker S.M. (2012). Initial assessment and management of pediatric trauma patients. Int. J. Crit. Illn. Inj. Sci..

[21] DeRoss A.L., Vane D.W. (2004). Early evaluation and resuscitation of the pediatric trauma patient. Semin. Pediatr. Surg..

[22] Magoteaux S.R., Notrica D.M., Langlais C.S., Linnaus M.E., Raines A.R., Letton R.W., Alder A.C., Greenwell C., Eubanks J.W., Lawson K.A. (2017). Hypotension and the need for transfusion in pediatric blunt spleen and liver injury: An ATOMAC+ prospective study. J. Pediatr. Surg..

[23] Acker S.N., Ross J.T., Partrick D.A., Tong S., Bensard D.D. (2015). Pediatric specific shock index accurately identifies severely injured children. J. Pediatr. Surg..

[24] Linnaus M.E., Notrica D.M., Langlais C.S., St Peter S.D., Leys C.M., Ostlie D.J., Maxson R.T., Ponsky T., Tuggle D.W., Eubanks J.W. (2017). Prospective validation of the shock index pediatric-adjusted (SIPA) in blunt liver and spleen trauma: An ATOMAC+ study. J. Pediatr. Surg..

[25] Phillips R., Acker S., Shahi N., Shirek G., Meier M., Goldsmith A., Recicar J., Moulton S., Bensard D. (2020). The shock index, pediatric age-adjusted (SIPA) enhanced: Prehospital and emergency department SIPA values forecast transfusion needs for blunt solid organ injured children. Surgery.

[26] Phillips R., Meier M., Shahi N., Acker S., Reppucci M., Shirek G., Recicar J., Moulton S., Bensard D. (2021). Elevated pediatric age-adjusted shock-index (SIPA) in blunt solid organ injuries. J. Pediatr. Surg..

[27] Polites S.F., Moody S., Williams R.F., Kayton M.L., Alberto E.C., Burd R.S., Schroeppel T.J., Baerg J.E., Munoz A., Rothstein W.B. (2020). Timing and volume of crystalloid and blood products in pediatric trauma: An Eastern Association for the Surgery of Trauma multicenter prospective observational study. J. Trauma Acute Care Surg..

[28] Notrica D.M., Eubanks J.W., Tuggle D.W., Maxson R.T., Letton R.W., Garcia N.M., Alder A.C., Lawson K.A., St Peter S.D., Megison S. (2015). Nonoperative management of blunt liver and spleen injury in children: Evaluation of the ATOMAC guideline using GRADE. J. Trauma Acute Care Surg..

[29] Stevens J., Pickett K., Moore H., Reppucci M.L., Phillips R., Moulton S., Bensard D. (2022). Thrombelastography and transfusion patterns in severely injured pediatric trauma patients with blunt solid organ injuries. J. Trauma Acute Care Surg..

[30] Notrica D.M., Sussman B.L., Sayrs L.W., St Peter S.D., Maxson R.T., Alder A.C., Eubanks J.W., Johnson J.J., Ostlie D.J., Ponsky T. (2021). Early vasopressor administration in pediatric blunt liver and spleen injury: An ATOMAC+ study. J. Pediatr. Surg..

[31] Calder B.W., Vogel A.M., Zhang J., Mauldin P.D., Huang E.Y., Savoie K.B., Santore M.T., Tsao K., Ostovar-Kermani T.G., Falcone R.A. (2017). Focused assessment with sonography for trauma in children after blunt abdominal trauma: A multi-institutional analysis. J. Trauma Acute Care Surg..

[32] Menaker J., Blumberg S., Wisner D.H., Dayan P.S., Tunik M., Garcia M., Mahajan P., Page K., Monroe D., Borgialli D. (2014). Use of the focused assessment with sonography for trauma (FAST) examination and its impact on abdominal computed tomography use in hemodynamically stable children with blunt torso trauma. J. Trauma Acute Care Surg..

[33] Scaife E.R., Rollins M.D., Barnhart D.C., Downey E.C., Black R.E., Meyers R.L., Stevens M.H., Gordon S., Prince J.S., Battaglia D. (2013). The role of focused abdominal sonography for trauma (FAST) in pediatric trauma evaluation. J. Pediatr. Surg..

[34] Long M.K., Vohra M.K., Bonnette A., Parra P.D.V., Miller S.K., Ayub E., Wang H.E., Cardenas-Turanzas M., Gordon R., Ugalde I.T. (2022). Focused assessment with sonography for trauma in predicting early surgical intervention in hemodynamically unstable children with blunt abdominal trauma. J. Am. Coll. Emerg. Physicians Open.

[35] McGaha P., Motghare P., Sarwar Z., Garcia N.M., Lawson K.A., Bhatia A., Langlais C.S., Linnaus M.E., Maxson R.T., Eubanks J.W. (2019). Negative Focused Abdominal Sonography for Trauma examination predicts successful nonoperative management in pediatric solid organ injury: A prospective Arizona-Texas-Oklahoma-Memphis-Arkansas + Consortium study. J. Trauma Acute Care Surg..

[36] Bahrami-Motlagh H., Hajijoo F., Mirghorbani M., SalevatiPour B., Haghighimorad M. (2020). Test characteristics of focused assessment with sonography for trauma (FAST), repeated FAST, and clinical exam in prediction of intra-abdominal injury in children with blunt trauma. Pediatr. Surg. Int..

[37] Pegoraro F., Giusti G., Giacalone M., Parri N. (2022). Contrast-enhanced ultrasound in pediatric blunt abdominal trauma: A systematic review. J. Ultrasound.

[38] Zhang Z., Hong Y., Liu N., Chen Y. (2017). Diagnostic accuracy of contrast enhanced ultrasound in patients with blunt abdominal trauma presenting to the emergency department: A systematic review and meta-analysis. Sci. Rep..

[39] Deftereos S.P., Foutzitzi S., Skarentzos K., Aggelidou M., Oikonomou P., Kambouri K. (2022). Role of Contrast Enhanced Ultrasound (CEUS) in the Paediatric Population with Blunt Abdominal Trauma: A Prospective Study from a Single Center Experience for Paediatric Blunt Abdominal Trauma. Maedica.

[40] Bowen D.K., Back S.J., Van Batavia J.P., Darge K., Long C.J., Weiss D.A. (2020). Does contrast-enhanced ultrasound have a role in evaluation and management of pediatric renal trauma? A preliminary experience. J. Pediatr. Surg..

[41] Paltiel H.J., Barth R.A., Bruno C., Chen A.E., Deganello A., Harkanyi Z., Henry M.K., Kljucevsek D., Back S.J. (2021). Contrast-enhanced ultrasound of blunt abdominal trauma in children. Pediatr. Radiol..

[42] Richards J.R., Knopf N.A., Wang L., McGahan J.P. (2002). Blunt abdominal trauma in children: Evaluation with emergency US. Radiology.

[43] The Royal College of Radiologists. Paediatric Trauma Protocols. https://www.rcr.ac.uk/media/k0blz5k3/rcr-publications_paediatric-tauma-protocols_august-2014.pdf.

[44] Streck C.J., Jewett B.M., Wahlquist A.H., Gutierrez P.S., Russell W.S. (2012). Evaluation for intra-abdominal injury in children after blunt torso trauma: Can we reduce unnecessary abdominal computed tomography by utilizing a clinical prediction model?. J. Trauma Acute Care Surg..

[45] Holmes J.F., Lillis K., Monroe D., Borgialli D., Kerrey B.T., Mahajan P., Adelgais K., Ellison A.M., Yen K., Atabaki S. (2013). Identifying children at very low risk of clinically important blunt abdominal injuries. Ann. Emerg. Med..

[46] Streck C.J., Vogel A.M., Zhang J., Huang E.Y., Santore M.T., Tsao K., Falcone R.A., Dassinger M.S., Russell R.T., Blakely M.L. (2017). Identifying Children at Very Low Risk for Blunt Intra-Abdominal Injury in Whom CT of the Abdomen Can Be Avoided Safely. J. Am. Coll. Surg..

[47] Arbra C.A., Vogel A.M., Plumblee L., Zhang J., Mauldin P.D., Dassinger M.S., Russell R.T., Blakely M.L., Streck C.J. (2018). External validation of a five-variable clinical prediction rule for identifying children at very low risk for intra-abdominal injury after blunt abdominal trauma. J. Trauma Acute Care Surg..

[48] Gaffley M., Neff L.P., Sieren L.M., Zeller K.A., Pranikoff T., Rush T., Petty J.K. (2021). Evaluation of an evidence-based guideline to reduce CT use in the assessment of blunt pediatric abdominal trauma. J. Pediatr. Surg..

[49] Boudiab E., Kawak S., Tom A., Studzinski D., Novotny N., Brahmamdam P., Akay B. (2022). Prospective evaluation of an evidence-based decision tool to assess pediatric blunt abdominal trauma (BAT). Pediatr. Surg. Int..

[50] Ozcan A., Ahn T., Akay B., Menoch M. (2022). Imaging for Pediatric Blunt Abdominal Trauma With Different Prediction Rules: Is the Outcome the Same?. Pediatr. Emerg. Care.

[51] Karam O., Sanchez O., Chardot C., La Scala G. (2009). Blunt abdominal trauma in children: A score to predict the absence of organ injury. J. Pediatr..

[52] Erlich T., Kitrey N.D. (2018). Renal trauma: The current best practice. Ther. Adv. Urol..

[53] Herman R., Guire K.E., Burd R.S., Mooney D.P., Ehlrich P.F. (2011). Utility of amylase and lipase as predictors of grade of injury or outcomes in pediatric patients with pancreatic trauma. J. Pediatr. Surg..

[54] Rosenfeld E.H., Vogel A., Russell R.T., Maizlin I., Klinkner D.B., Polites S., Gaines B., Leeper C., Anthony S., Waddell M. (2018). Comparison of diagnostic imaging modalities for the evaluation of pancreatic duct injury in children: A multi-institutional analysis from the Pancreatic Trauma Study Group. Pediatr. Surg. Int..

[55] Zimmermann P., Schmidt T., Nelson J., Gosemann J.H., Bassler S., Stahmeyer J.T., Hirsch F.W., Lacher M., Zeidler J. (2020). Pediatric solid organ injury—Frequency of abdominal imaging is determined by the treating department. Medicine.

[56] Mehall J.R., Ennis J.S., Saltzman D.A., Chandler J.C., Grewal H., Wagner C.W., Jackson R.J., Smith S.D. (2001). Prospective results of a standardized algorithm based on hemodynamic status for managing pediatric solid organ injury. J. Am. Coll. Surg..

[57] McVay M.R., Kokoska E.R., Jackson R.J., Smith S.D. (2008). Throwing out the “grade” book: Management of isolated spleen and liver injury based on hemodynamic status. J. Pediatr. Surg..

[58] Katz M.G., Kastenberg Z.J., Taylor M.A., Bolinger C.D., Scaife E.R., Fenton S.J., Russell K.W. (2019). Reduction of resource utilization in children with blunt solid organ injury. J. Pediatr. Surg..

[59] Stewart S., Fraser J.A., Rentea R.M., Aguayo P., Juang D., Fraser J.D., Snyder C.L., Hendrickson R.J., St Peter S.D., Oyetunji T.A. (2023). Institutional outcomes of blunt liver and splenic injury in the Arizona-Texas-Oklahoma-Memphis-Arkansas Consortium era. J. Trauma Acute Care Surg..

[60] Mehl S.C., Cunningham M.E., Streck C.J., Pettit R., Huang E.Y., Santore M.T., Tsao K., Falcone R.A., Dassinger M.S., Haynes J.H. (2022). Characteristics and predictors of intensive care unit admission in pediatric blunt abdominal trauma. Pediatr. Surg. Int..

[61] Derderian S.C., Meier M., Bensard D.D., Partrick D.A., Acker S.N. (2022). Adolescent blunt solid organ injury: Differences in management strategies and outcomes between pediatric and adult trauma centers. Am. J. Surg..

[62] Golden J., Mitchell I., Kuzniewski S., Lipskar A., Prince J.M., Bank M., Stylianos S., Rosen N.G. (2014). Reducing scheduled phlebotomy in stable pediatric patients with blunt liver or spleen injury. J. Pediatr. Surg..

[63] Denning N.L., Abd El-Shafy I., Munoz A., Vannix I., Hazboun R., Luo-Owen X., Cordova J.F., Baerg J., Cullinane D.C., Prince J.M. (2019). Safe phlebotomy reduction in stable pediatric liver and spleen injuries. J. Pediatr. Surg..

[64] Acker S.N., Petrun B., Partrick D.A., Roosevelt G.E., Bensard D.D. (2015). Lack of utility of repeat monitoring of hemoglobin and hematocrit following blunt solid organ injury in children. J. Trauma Acute Care Surg..

[65] Stottlemyre R.L., Notrica D.M., Cohen A.S., Sayrs L.W., Naiditch J., St Peter S.D., Leys C.M., Ostlie D.J., Maxson R.T., Ponsky T. (2023). Hemodilution in pediatric trauma: Defining the expected hemoglobin changes in patients with liver and/or spleen injury: An ATOMAC+ secondary analysis. J. Pediatr. Surg..

[66] Welker E., Novak J., Jelsma L., Koehler T., Davis A., DeCou J., Durkin E. (2018). Continuous hemoglobin monitoring in pediatric trauma patients with solid organ injury. J. Pediatr. Surg..

[67] Ryan M.L., Cairo S.B., McLaughlin C., Herring L., Williams R.F. (2023). Utility of continuous pulse CO-oximetry for hemoglobin monitoring in pediatric patients with solid organ injuries at level 1 trauma centers: A pilot study. J. Trauma Acute Care Surg..

[68] Ryan M.L., Maxwell A.C., Manning L., Jacobs J.D., Bachier-Rodriguez M., Feliz A., Williams R.F. (2016). Noninvasive hemoglobin measurement in pediatric trauma patients. J. Trauma Acute Care Surg..

[69] LeeVan E., Zmora O., Cazzulino F., Burke R.V., Zagory J., Upperman J.S. (2016). Management of pediatric blunt renal trauma: A systematic review. J. Trauma Acute Care Surg..

[70] O’Neill J.A. (2000). Advances in the management of pediatric trauma. Am. J. Surg..

[71] Spijkerman R., Bulthuis L.C.M., Hesselink L., Nijdam T.M.P., Leenen L.P.H., de Bruin I. (2021). Management of pediatric blunt abdominal trauma in a Dutch level one trauma center. Eur. J. Trauma Emerg. Surg..

[72] Basaran A., Ozkan S. (2019). Evaluation of intra-abdominal solid organ injuries in children. Acta Biomed..

[73] Stylianos S., Egorova N., Guice K.S., Arons R.R., Oldham K.T. (2006). Variation in treatment of pediatric spleen injury at trauma centers versus nontrauma centers: A call for dissemination of American Pediatric Surgical Association benchmarks and guidelines. J. Am. Coll. Surg..

[74] Hsiao M., Sathya C., de Mestral C., Langer J.C., Gomez D., Nathens A.B. (2014). Population-based analysis of blunt splenic injury management in children: Operative rate is an informative quality of care indicator. Injury.

[75] Safavi A., Skarsgard E.D., Rhee P., Zangbar B., Kulvatunyou N., Tang A., O’Keeffe T., Friese R.S., Joseph B. (2016). Trauma center variation in the management of pediatric patients with blunt abdominal solid organ injury: A national trauma data bank analysis. J. Pediatr. Surg..

[76] Lippert S.J., Hartin C.W., Ozgediz D.E., Glick P.L., Caty M.G., Flynn W.J., Bass K.D. (2013). Splenic conservation: Variation between pediatric and adult trauma centers. J. Surg. Res..

[77] Yung N., Solomon D., Schuster K., Christison-Lagay E. (2020). Closing the gap in care of blunt solid organ injury in children. J. Trauma Acute Care Surg..

[78] Linnaus M.E., Langlais C.S., Garcia N.M., Alder A.C., Eubanks J.W., Maxson R.T., Letton R.W., Ponsky T.A., St Peter S.D., Leys C. (2017). Failure of nonoperative management of pediatric blunt liver and spleen injuries: A prospective Arizona-Texas-Oklahoma-Memphis-Arkansas Consortium study. J. Trauma Acute Care Surg..

[79] Notrica D.M., Sayrs L.W., Bhatia A., Letton R.W., Alder A., St Peter S., Ponsky T.A., Eubanks J.W., Lawson K.A., Ostlie D.J. (2018). The incidence of delayed splenic bleeding in pediatric blunt trauma. J. Pediatr. Surg..

[80] Holmes J.H., Wiebe D.J., Tataria M., Mattix K.D., Mooney D.P., Scaife E.R., Brown R.L., Groner J.I., Brundage S.I., Tres Scherer L.R. (2005). The failure of nonoperative management in pediatric solid organ injury: A multi-institutional experience. J. Trauma.

[81] Nance M.L., Holmes J.H., Wiebe D.J. (2006). Timeline to operative intervention for solid organ injuries in children. J. Trauma..

[82] Arbra C.A., Vogel A.M., Zhang J., Mauldin P.D., Huang E.Y., Savoie K.B., Santore M.T., Tsao K., Ostovar-Kermani T.G., Falcone R.A. (2017). Acute procedural interventions after pediatric blunt abdominal trauma: A prospective multicenter evaluation. J. Trauma Acute Care Surg..

[83] Sakamoto R., Matsushima K., de Roulet A., Beetham K., Strumwasser A., Clark D., Inaba K., Demetriades D. (2018). Nonoperative management of penetrating abdominal solid organ injuries in children. J. Surg. Res..

[84] Parrado R., Notrica D.M., Garcia N.M., Alder A.C., Eubanks J.W., Maxson R.T., Letton R.W., Ponsky T.A., St Peter S.D., Leys C. (2019). Use of Laparoscopy in Pediatric Blunt and Spleen Injury: An Unexpectedly Common Procedure After Cessation of Bleeding. J. Laparoendosc. Adv. Surg. Tech. A.

[85] Swendiman R.A., Goldshore M.A., Blinman T.A., Nance M.L. (2019). Laparoscopic Management of Pediatric Abdominal Trauma: A National Trauma Data Bank Experience. J. Laparoendosc. Adv. Surg. Tech. A.

[86] Wisner D.H., Kuppermann N., Cooper A., Menaker J., Ehrlich P., Kooistra J., Mahajan P., Lee L., Cook L.J., Yen K. (2015). Management of children with solid organ injuries after blunt torso trauma. J. Trauma Acute Care Surg..

[87] van As A.B., Millar A.J. (2017). Management of paediatric liver trauma. Pediatr. Surg. Int..

[88] Duron V., Stylianos S. (2020). Strategies in liver Trauma. Semin. Pediatr. Surg..

[89] Stalker H.P., Kaufman R.A., Towbin R. (1986). Patterns of liver injury in childhood: CT analysis. AJR Am. J. Roentgenol..

[90] Arbuthnot M., Onwubiko C., Mooney D. (2016). The lost art of the splenorrhaphy. J. Pediatr. Surg..

[91] Keller M.S., Sartorelli K.H., Vane D.W. (1996). Associated head injury should not prevent nonoperative management of spleen or liver injury in children. J. Trauma.

[92] Resende V., Petroianu A. (2003). Functions of the splenic remnant after subtotal splenectomy for treatment of severe splenic injuries. Am. J. Surg..

[93] Tahir F., Ahmed J., Malik F. (2020). Post-splenectomy Sepsis: A Review of the Literature. Cureus.

[94] Di Sabatino A., Carsetti R., Corazza G.R. (2011). Post-splenectomy and hyposplenic states. Lancet.

[95] Murphy G.P., Gaither T.W., Awad M.A., Osterberg E.C., Baradaran N., Copp H.L., Breyer B.N. (2017). Management of Pediatric Grade IV Renal Trauma. Curr. Urol. Rep..

[96] Serafetinides E., Kitrey N.D., Djakovic N., Kuehhas F.E., Lumen N., Sharma D.M., Summerton D.J. (2015). Review of the current management of upper urinary tract injuries by the EAU Trauma Guidelines Panel. Eur. Urol..

[97] Farr B.J., Armstrong L.B., Barnett S.C., Mooney D.P. (2022). Variation in management of pediatric post-traumatic urine leaks. Eur. J. Trauma Emerg. Surg..

[98] Ghani M.O.A., Snyder E., Xu M.C., McKay K.G., Foster J., Tong C., Clayton D.B., Greeno A., Azam B., Zhao S. (2022). Urine leaks in children sustaining blunt renal trauma. J. Trauma Acute Care Surg..

[99] Jabbour G., Lombardo G., Litow K., Prabhakaran K., Carroll F., Policastro A., Rhee P., Latifi R. (2020). Bilateral Renal Artery Occlusion after Blunt Abdominal Trauma. Am. Surg..

[100] Vidal E., Marrone G., Gasparini D., Pecile P. (2011). Radiological treatment of renal artery occlusion after blunt abdominal trauma in a pediatric patient: Is it never too late?. Urology.

[101] Omura T., Matsushita K., Arase M., Yagi T. (2019). Three cases of paediatric pancreatic injury involving the main pancreatic duct. Trauma Case Rep..

[102] Rosenfeld E.H., Vogel A.M., Klinkner D.B., Escobar M., Gaines B., Russell R., Campbell B., Wills H., Stallion A., Juang D. (2017). The utility of ERCP in pediatric pancreatic trauma. J. Pediatr. Surg..

[103] Gong S.C., An S., Shin I.S., Jung P.Y. (2023). Usefulness of Endoscopic Retrograde Cholangiopancreatography in the Diagnosis and Treatment of Traumatic Pancreatic Injury in Children. Diagnostics.

[104] Stundner-Ladenhauf H.N., Bauer L., Heil C., Holzinger J., Stundner O., Metzger R. (2022). Minimally Invasive Approaches for Traumatic Rupture of the Pancreas in Children-A Case Series. Children.

[105] Ishikawa M., Shimojima N., Koyama T., Miyaguni K., Tsukizaki A., Mizuno Y., Hashimoto M., Ishihama H., Tomita H., Shimotakahara A. (2021). Efficacy of early endoscopic intervention in pediatric pancreatic duct injury management. Pediatr. Surg. Int..

[106] Mora M.C., Wong K.E., Friderici J., Bittner K., Moriarty K.P., Patterson L.A., Gross R.I., Tirabassi M.V., Tashjian D.B. (2016). Operative vs Nonoperative Management of Pediatric Blunt Pancreatic Trauma: Evaluation of the National Trauma Data Bank. J. Am. Coll. Surg..

[107] Linnaus M.E., Notrica D.M. (2016). Evaluating the Outcomes of Operative vs Nonoperative Management of Pediatric Blunt Pancreatic Trauma. J. Am. Coll. Surg..

[108] Iqbal C.W., St Peter S.D., Tsao K., Cullinane D.C., Gourlay D.M., Ponsky T.A., Wulkan M.L., Adibe O.O., Pancreatic Trauma in Children Study Group (2014). Operative vs nonoperative management for blunt pancreatic transection in children: Multi-institutional outcomes. J. Am. Coll. Surg..

[109] Goldberg-Murow M., Steiner Z., Lakovsky Y., Dlugy E., Baazov A., Freud E., Samuk I. (2021). Blunt High-Grade Pancreatic Injury in Children: A 20-Year Experience in Two Pediatric Surgical Centers. Isr. Med. Assoc. J..

[110] Everson E., Buschel H., Carroll J., Palamuthusingam P. (2023). Paediatric pancreatic trauma in North Queensland: A 10-year retrospective review. BMC Pediatr..

[111] Edwards M.J., Crudo D.F., Carlson T.L., Pedersen A.M., Keller L. (2013). Pancreatic atrophy and diabetes mellitus following blunt abdominal trauma. J. Pediatr. Surg..

[112] Catellani B., Caracciolo D., Magistri P., Guidetti C., Menduni N., Yu H., Odorizzi R., Guerrini G.P., Ballarin R., Di Sandro S. (2023). Laparoscopic Management of Blunt Pancreatic Trauma in Adults and Pediatric Patients: A Systematic Review. Biomed. Res. Int..

[113] Tyberg A., Karia K., Gabr M., Desai A., Doshi R., Gaidhane M., Sharaiha R.Z., Kahaleh M. (2016). Management of pancreatic fluid collections: A comprehensive review of the literature. World J. Gastroenterol..

[114] Rosenfeld E.H., Vogel A.M., Jafri M., Burd R., Russell R., Beaudin M., Sandler A., Thakkar R., Falcone R.A., Wills H. (2019). Management and outcomes of peripancreatic fluid collections and pseudocysts following non-operative management of pancreatic injuries in children. Pediatr. Surg. Int..

[115] Ingram M.C., Siddharthan R.V., Morris A.D., Hill S.J., Travers C.D., McKracken C.E., Heiss K.F., Raval M.V., Santore M.T. (2016). Hepatic and splenic blush on computed tomography in children following blunt abdominal trauma: Is intervention necessary?. J. Trauma Acute Care Surg..

[116] Bansal S., Karrer F.M., Hansen K., Partrick D.A. (2015). Contrast blush in pediatric blunt splenic trauma does not warrant the routine use of angiography and embolization. Am. J. Surg..

[117] Leung-Tack M., Ong E.G.P., McGuirk S. (2022). Interventional radiology and open surgery: An effective partnership for solid organ trauma. J. Pediatr. Surg..

[118] Swendiman R.A., Goldshore M.A., Fenton S.J., Nance M.L. (2020). Defining the role of angioembolization in pediatric isolated blunt solid organ injury. J. Pediatr. Surg..

[119] Yanchar N.L., Lockyer L., Ball C.G., Assen S. (2021). Pediatric versus adult paradigms for management of adolescent injuries within a regional trauma system. J. Pediatr. Surg..

[120] Dantes G., Kolousek A., Doshi N., Dutreuil V., Sciarretta J.D., Sola R., Shah J., Smith R.N., Smith A.D., Koganti D. (2024). Utilization of Angiography in Pediatric Blunt Abdominal Injury at Adult versus Pediatric Trauma Centers. J. Surg. Res..

[121] Skattum J., Gaarder C., Naess P.A. (2014). Splenic artery embolisation in children and adolescents—An 8 year experience. Injury.

[122] St Peter S.D., Sharp S.W., Snyder C.L., Sharp R.J., Andrews W.S., Murphy J.P., Islam S., Holcomb G.W., Ostlie D.J. (2011). Prospective validation of an abbreviated bedrest protocol in the management of blunt spleen and liver injury in children. J. Pediatr. Surg..

[123] St Peter S.D., Aguayo P., Juang D., Sharp S.W., Snyder C.L., Holcomb G.W., Ostlie D.J. (2013). Follow up of prospective validation of an abbreviated bedrest protocol in the management of blunt spleen and liver injury in children. J. Pediatr. Surg..

[124] Graziano K.D., Juang D., Notrica D., Grandsoult V.L., Acosta J., Sharp S.W., Murphy J.P., St Peter S.D. (2014). Prospective observational study with an abbreviated protocol in the management of blunt renal injury in children. J. Pediatr. Surg..

[125] Notrica D.M., Sayrs L.W., Krishna N., Ostlie D.J., Letton R.W., Alder A.C., St Peter S.D., Ponsky T.A., Eubanks J.W., Tuggle D.W. (2019). Adherence to APSA activity restriction guidelines and 60-day clinical outcomes for pediatric blunt liver and splenic injuries (BLSI). J. Pediatr. Surg..

[126] Podda M., De Simone B., Ceresoli M., Virdis F., Favi F., Wiik Larsen J., Coccolini F., Sartelli M., Pararas N., Beka S.G. (2022). Follow-up strategies for patients with splenic trauma managed non-operatively: The 2022 World Society of Emergency Surgery consensus document. World J. Emerg. Surg..

[127] Stokes S.C., Brown E.G., Jackson J.E., Leshikar D.E., Stephenson J.T. (2021). Implementation of an evidence-based accelerated pathway: Can hospital length of stay for children with blunt solid organ injury be safely decreased?. Pediatr. Surg. Int..

[128] Evans L.L., Williams R.F., Jin C., Plumblee L., Naik-Mathuria B., Streck C.J., Jensen A.R. (2021). Hospital-based intervention is rarely needed for children with low-grade blunt abdominal solid organ injury: An analysis of the Trauma Quality Improvement Program registry. J. Trauma Acute Care Surg..

[129] Plumblee L., Williams R., Vane D., Zhang J., Jensen A., Naik-Mathuria B., Evans L., Streck C.J. (2020). Isolated low-grade solid organ injuries in children following blunt abdominal trauma: Is it time to consider discharge from the emergency department?. J. Trauma Acute Care Surg..

[130] Butt E., Kotagal M., Shebesta K., Bailey A., Moody S., Falcone R. (2021). Admission for Isolated Low-Grade Solid Organ Injury May Not Be Necessary in Pediatric Patients. J. Trauma. Nurs..

[131] Notrica D.M., Sussman B.L., Garcia N.M., Leys C.M., Maxson R.T., Bhatia A., Letton R.W., Ponsky T., Lawson K.A., Eubanks J.W. (2019). Reimaging in pediatric blunt spleen and liver injury. J. Pediatr. Surg..

[132] Durkin N., Deganello A., Sellars M.E., Sidhu P.S., Davenport M., Makin E. (2016). Post-traumatic liver and splenic pseudoaneurysms in children: Diagnosis, management, and follow-up screening using contrast enhanced ultrasound (CEUS). J. Pediatr. Surg..

[133] Letton R.W., Campbell B.T., Falcone R.A., Gaines B.A., Gourlay D.M., Groner J.I., Mooney D.P., Nance M.L., Notrica D.M., Petty J.K. (2017). Letter to the Editor: “Post-traumatic liver and splenic pseudoaneurysms in children: Diagnosis, management, and follow-up screening using contrast enhanced ultrasound (CEUS)” by Durkin et al J Pediatr Surg 51 (2016) 289-292. J. Pediatr. Surg..

[134] Martin K., Vanhouwelingen L., Butter A. (2011). The significance of pseudoaneurysms in the nonoperative management of pediatric blunt splenic trauma. J. Pediatr. Surg..

[135] Schellenberg M., Emigh B., Nichols C., Dilday J., Ugarte C., Onogawa A., Shapiro D., Im D.D., Inaba K. (2023). Pseudoaneurysm Screening after Pediatric High Grade Solid Organ Injury. Am. Surg..

[136] Perea L.L., Echeverria Rosario K., Staman S., Fox N. (2022). Pediatric Trauma: What Hurts?. Pediatr. Emerg. Care.

[137] Yanchar N., Tardif P.A., Freire G., Berube M., Stelfox H.T., Beaudin M., Stang A., Beno S., Weiss M., Labrosse M. (2023). Clinical practice guideline recommendations for pediatric solid organ injury care: A Systematic Review. J. Trauma Acute Care Surg..

[138] Georgeades C., Van Arendonk K., Gourlay D. (2021). Venous thromboembolism prophylaxis after pediatric trauma. Pediatr. Surg. Int..

[139] Alexander M., Zaghal A., Wetjen K., Shelton J., Shilyansky J. (2019). Pediatric trauma center verification improves quality of care and reduces resource utilization in blunt splenic injury. J. Pediatr. Surg..

[140] Vogel A.M., Zhang J., Mauldin P.D., Williams R.F., Huang E.Y., Santore M.T., Tsao K., Falcone R.A., Dassinger M.S., Haynes J.H. (2019). Variability in the evalution of pediatric blunt abdominal trauma. Pediatr. Surg. Int..

[141] Shahi N., Shahi A.K., Phillips R., Shirek G., Bensard D., Moulton S.L. (2021). Decision-making in pediatric blunt solid organ injury: A deep learning approach to predict massive transfusion, need for operative management, and mortality risk. J. Pediatr. Surg..

